# *MouseMove*: an open source program for semi-automated analysis of movement and cognitive testing in rodents

**DOI:** 10.1038/srep16171

**Published:** 2015-11-04

**Authors:** Andre L. Samson, Lining Ju, Hyun Ah Kim, Shenpeng R. Zhang, Jessica A. A. Lee, Sharelle A. Sturgeon, Christopher G. Sobey, Shaun P. Jackson, Simone M. Schoenwaelder

**Affiliations:** 1 Australian Centre for Blood Diseases, Alfred Medical Research and Education Precinct (AMREP), Monash University, Melbourne, 3004; 2 Heart Research Institute, and Charles Perkins Centre, The University of Sydney, Camperdown, NSW 2006, Australia; 3 Department of Pharmacology, Monash University, Clayton 3800, Victoria

## Abstract

The Open Field (OF) test is one of the most commonly used assays for assessing exploratory behaviour and generalised locomotor activity in rodents. Nevertheless, the vast majority of researchers still rely upon costly commercial systems for recording and analysing OF test results. Consequently, our aim was to design a freely available program for analysing the OF test and to provide an accompanying protocol that was minimally invasive, rapid, unbiased, without the need for specialised equipment or training. Similar to commercial systems, we show that our software—called *MouseMove*—accurately quantifies numerous parameters of movement including travel distance, speed, turning and curvature. To assess its utility, we used *MouseMove* to quantify unilateral locomotor deficits in mice following the filament-induced middle cerebral artery occlusion model of acute ischemic stroke. *MouseMove* can also monitor movement within defined regions-of-interest and is therefore suitable for analysing the Novel Object Recognition test and other field-related cognitive tests. To the best of our knowledge, *MouseMove* is the first open source software capable of providing qualitative and quantitative information on mouse locomotion in a semi-automated and high-throughput fashion, and hence *MouseMove* represents a sound alternative to commercial movement analysis systems.

The Open Field (OF) test is amongst the most commonly used assays for monitoring exploratory behaviour and locomotor activity in laboratory animals^1^. The OF test relies on the principle that rodents and other laboratory animals will innately explore novel surroundings. Ideally, the OF should be devoid of visual landmarks, olfactory cues and be situated in a quiet dimly lit room; so as to encourage exploration and ambulation, to minimise learning/memory triggers and to reduce stress on the laboratory animal during testing. It is because of these simple underlying principles and low animal handling requirements that the OF test represents a relatively quick, reproducible and robust assay. Accordingly, the OF test has been used to assess neurological effects across a wide array of experimental paradigms, including acquired brain injury^2^, psychostimulant administration^3^, stress/anxiety induction^4^, aging^5^, gender^5^, circadian cycling^6^, differing genophenotypic backgrounds^7^ and environmental factors^8^.

Despite its widespread popularity, the vast majority of research groups rely upon commercial systems for recording and digitally analysing the OF test. These commercial system use an overhead video camera or a laser-gridded arena, and quantify movement parameters using proprietary software. Examples of current commercial systems capable of performing OF testing include the ANY-maze (Stoelting Co; IL, USA), Ethovision® XT (Noldus; Wageningen, The Netherlands), TopScan (Clever Sys Inc.; VA, USA), and Opto-Varimex (Columbus Instruments; OH, USA). While these commercial systems are excellent in their recording and analytical capability, they are relatively expensive, offer little methodological transparency or flexibility and often restrict OF testing to laboratories with the financial means to establish specialised behavioural suites.

Numerous open source programs have been released as alternatives to the commercial OF systems^9^,^10^,^11^,^12^,^13^. All but one of these prior programs restricts its analyses to two main aspects of movement: travel distance and time spent within a defined region-of-interest (ROI). The most recent freeware, *EthoWatcher*, represents a significant improvement in that it allows users to analyse a wider array of rodent movement parameters such as grooming and rearing behaviours^13^. This being said, many other routine aspects of movement, including stationary fraction, object speed, laterality differences or ROI time, in combination with capacity for batch analysis, are currently not addressed by existing programs. As a result, commercial systems still offer vastly superior performance and throughput for OF test analysis.

Here we describe *MouseMove*—an open source program that offers semi-automated and high-throughput analysis of a wide array of movement parameters, including distance travelled, mean speed, speed variance, stationary fraction and laterality (i.e. left/right turn, turning offset and curvature). Using a model of acquired brain injury (the transient middle cerebral artery occlusion [MCAo] model of experimental stroke), which is well known to cause altered locomotion^14^,^15^, we show that *MouseMove* can discern both quantitative and qualitative differences in movement across an OF. Importantly, using a mechanical calibration system, we demonstrate that *MouseMove* tracks changes in distance, speed and laterality with >96% accuracy. *MouseMove* also has a region-of-interest function which allows quantitative analysis of cognitive tests such as the Novel Object Recognition (NOR) test. Taken together, *MouseMove* represents a sound and freely available alternative to commercial platforms for analysing arena-related assays such as the OF test and the NOR test.

## Results

### Design of an OF, automated image processing and movement analysis using *MouseMove*

**Figure 1:**
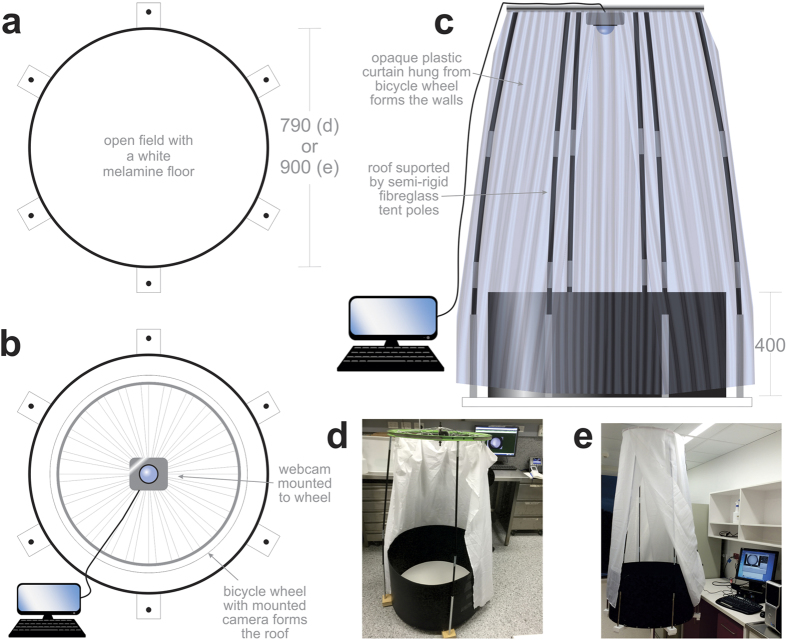
Design of the OFs used for testing *MouseMove*. Aerial perspective scaled drawing of our OF without (**a**) or with (**b**) its roof. (**c)** Side perspective scaled drawing of our OF. (**d**,**e**) Photos of OF #1 (**d**) and OF #2 (**e**) used to test *MouseMove*.

**Figure 2:**
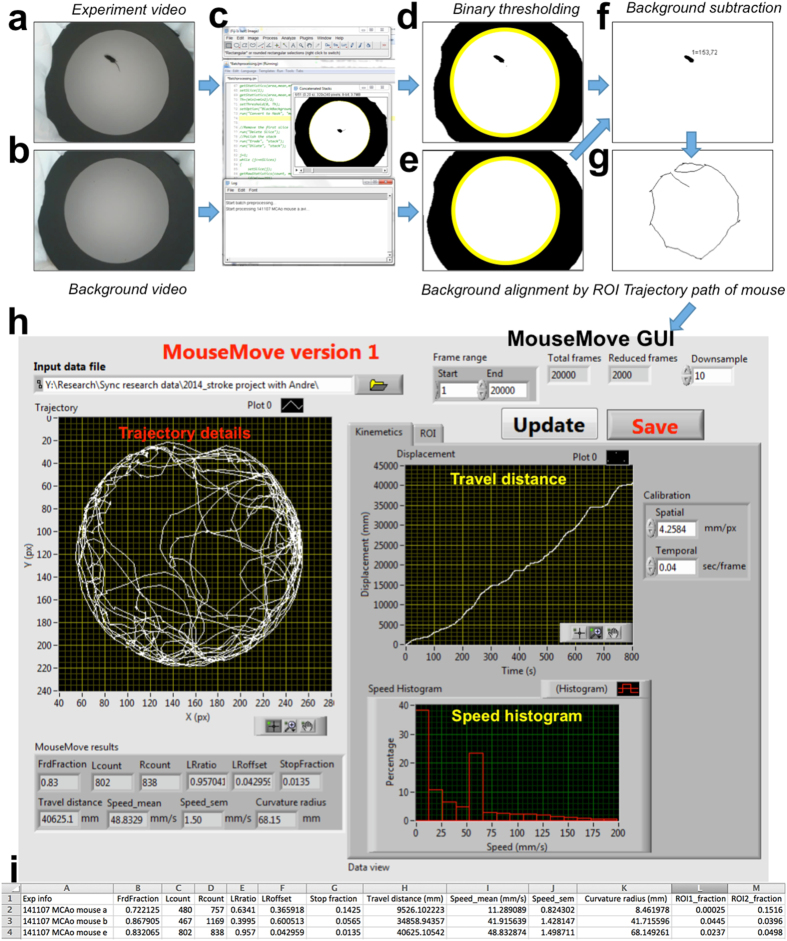
Stepwise image processing of an OF test by *MouseMove*. (**a**) Video frame of a mouse during OF testing. (**b**) Video frame of the empty OF. (**c**) Screenshot of *MouseMove*’s preprocessing macro into which the two videos are entered. (**d**) Same frame as in ‘Panel (**a**)’ but after binary thresholding. (**e**) Same frame as in ‘Panel (**b**)’ but after binary thresholding. The yellow rings in ‘Panels (**d**,**e**)’ indicate the perimeter of the OF as detected by *MouseMove*, which allows automatic spatial alignment of the two videos. (**f**) Same frame from ‘Panel (**a**)’ but after subtraction of ‘Panel (**e**)’ from ‘Panel (**d**)’. ‘1 = 153,72’ stipulates the assigned number of the measurable object (i.e. one) and its arbitrary *x,y* coordinates at that point in time (i.e. x = 153 and y = 72). (**g**) Cumulative trajectories of the same mouse over 100 s in the OF. (**h**) Screenshot of *MouseMove*’s graphical user interface (GUI) and visual depiction of the analyses performed by *MouseMove* for an OF test. (**i**) Example of *MouseMove*’s text file output for the OF testing of three representative MCAo-operated mice.

An OF arena was first assembled to test *MouseMove*. As shown in Fig. 1, the OF had a circular white melamine floor, black plastic walls, was surrounded by a white polyester curtain and had a webcam positioned above its centre. For each OF test, a mouse is placed in the centre of the arena and video footage captured (‘experiment video’, Fig. 2a; refer to *Methods* for details). Directly after testing, a ~10 second video of the empty arena is captured (‘background video’, Fig. 2b). The open source program, ImageJ, is then initialised and the ‘experiment’ and ‘background’ videos are uploaded (Fig. 2c) using our ImageJ macro *Preprocessing*.ijm (Supplementary File 1). The macro automatically performs the following stepwise processes: Step 1—thresholding of the raw videos to create binary videos. Step 2—*x,y* alignment between the experiment and background videos (Fig. 2d,e). Step 3—subtraction of the aligned background video from the experiment video (Fig. 2f). Step 4—object recognition and trajectory generation using the published MTrack2 plugin^16^. The macro generates a saveable image of the mouse’s cumulative trajectories (Fig. 2g) and a text file stipulating the *x,y* coordinates of the mouse over time. Next, *MouseMove*’s graphical user interface (GUI) is initialised. Pertinent parameters (video frame rate, range of frames to be analysed, spatial scale of the video) and the location of the macro-generated text file are entered using *MouseMove.*exe (Supplementary File 2) A detailed analysis of the movement patterns is then automatically performed, whereby *MouseMove* measures the fractional time spent stationary, distance travelled, speed mean/standard error of the mean (s.e.m.) and various indices of laterality (number of left turns, number of right turns, ratio of left:right turns (LRratio), turn offset and curvature radius). By default, the analytical results of *MouseMove* are depicted in both a visual/graphical form (Fig. 2h) and as a saveable text file (Fig. 2i).

### Mechanical calibration of *MouseMove*

**Figure 3:**
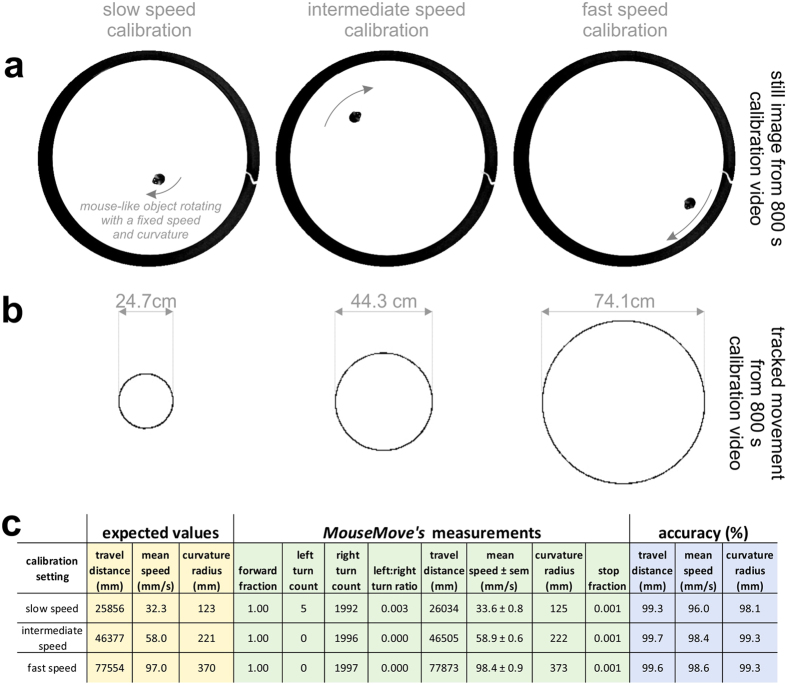
Mechanical calibration of *MouseMove*. (**a**) Video still image of a mouse-like object affixed to a perspex arm driven by a speed-controlled rotor. The clockwise rotation of the arm is indicated. The position of the mouse-like object was varied (n = 3 positions) to produce a slow, intermediate and fast circling object for tracking. (**b**) The corresponding cumulative *x,y* coordinates tracked by the preprocessing macro over the 800 s videos. The diameter of the tracked circular path for the mouse-like object is indicated. (**c**) The tracked *x,y* coordinates (from panel (**b**)) were fed into *MouseMove*’s GUI and the observed measurements (green shading), the expected values (yellow shading), and the percent accuracy between the observed versus expected measurements (blue shading) is shown.

To calibrate *MouseMove* we took video footage of a mouse-shaped object affixed to a perspex arm being driven by a speed-controlled rotor (Fig. 3a). We tested *MouseMove*’s tracking capabilities across a range of defined speeds and curvatures by varying the position of the mouse-shaped object along the perspex arm (Fig. 3b). Via this approach, we found that *MouseMove* quantified movement parameters such as distanced travelled, speed and curvature radius with >96% accuracy (Fig. 3c). Importantly, the calibration settings encompass the range of movement typically exhibited by adult mice (as determined by batch analysis of >150 mice; data not shown).

**Figure 4:**
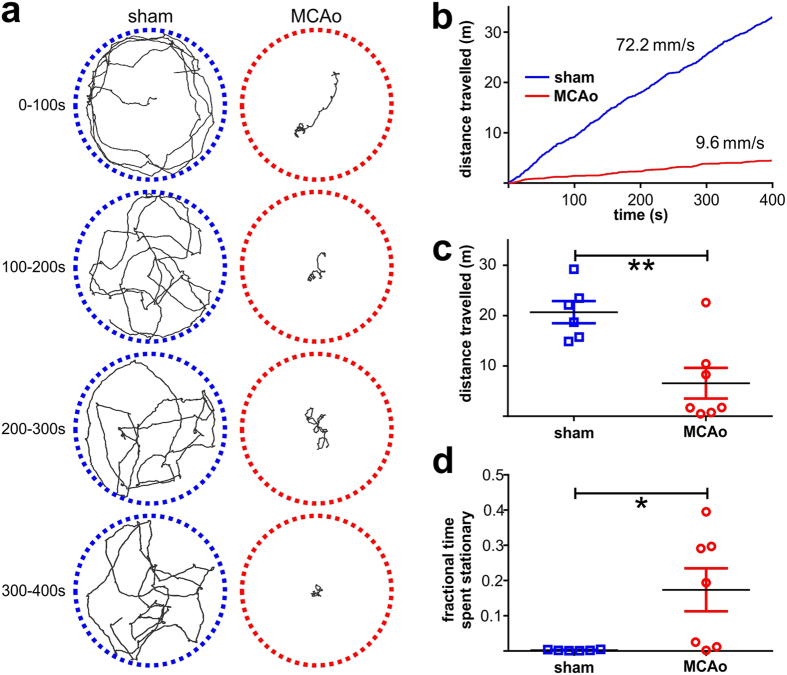
Analysis of locomotor activity in mice after experimental stroke using *MouseMove*. (**a**) Cumulative trajectories of a representative MCAo-operated mouse and a sham-operated mouse over 100 s intervals in the OF. (**b**) Plot of distance travelled over time for a representative sham- and MCAo-operated mouse in the OF. Numerical annotations indicate the mean speed for these same sham- and MCAo-operated mice over the 400 s period. (**c**,**d**) ‘Distance travelled’ (**c**) and ‘Fractional time spent stationary’ (**d**) for sham-operated (n = 6) and MCAo-operated (n = 7) mice over a 400 s period in the OF. Dot points represent the mean value for individual mice. Line and error bars represent the cohort mean ± s.e.m. **p < 0.01 and *p < 0.05 as determined by two-sided unpaired t-test without (**c**) or with Welch’s correction (**d**).

**Figure 5:**
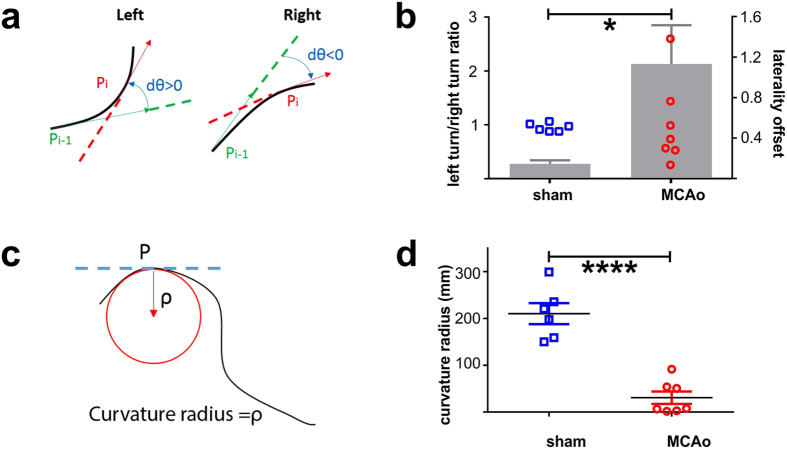
Analysis of laterality differences in mice after experimental stroke using *MouseMove*. (**a**) Schematics showing how *MouseMove* determines whether a mouse is turning left or right at each location (P_i_). The algorithm first determines the vector at P_i_ (red) and at a prior time point (P_i-1_; green). If the angle change from P_i-1_ to P_i_ is Δ*θ*_i_>0, then the mouse is turning left. If the angle change from P_i−1_ to P_i_ is Δ*θ*_i_ <0, then the mouse is turning right. (**b**) The ratio of left-turns versus right-turns (LRatio; dots plotted against the left-hand *y*-axis) and the laterality offset (the absolute value of 1-LRatio; bar graph plotted against the right-hand *y*-axis) for MCAo-operated (n = 7) and sham-operated (n = 6) mice over a 400  s period. ****p < 0.0001 as determined by two-sided unpaired t-test for the differences in laterality offset. (**c**) Schematic showing how *MouseMove* determines the trajectory at a point (P) and how the curvature radius refers to the inner tangential circle radius (red). (**d**) Trajectory curvature radius for sham-operated (n = 6) and MCAo-operated (n = 7) mice over a 400 s period. Dot points represent the mean value for individual mice. Line and error bars represent the cohort mean ± s.e.m. *p < 0.05 as determined by two-sided unpaired t-test. These results were derived from the same videos as were used in Fig. 4.

As an additional means with which to assess accuracy, we attempted to correlate the findings of *MouseMove* with that of *EthoWatcher* software for the speed-controlled rotor videos (Fig. 3) and for the videos of sham- or MCAo-operated mice (Figs 4 and 5). Unfortunately, this comparison could not be made due to inconsistencies in the object tracking function of *EthoWatcher* (data not shown).

### *MouseMove* analysis can be used to quantify reduced locomotor activity in mice after experimental stroke

To demonstrate the utility of *MouseMove* in an experimental setting, we used it to analyse the OF behaviour of mice that had undergone the MCAo model of acute ischaemic stroke, which is well known to produce focal unilateral cerebral infarction and impaired locomotor activity in rodents^14^,^15^,^17^. *MouseMove* highlighted both qualitative (Fig. 4a and Supplementary Video 1) and quantitative (Fig. 4b–d) differences in locomotor activity between sham-operated mice and MCAo-operated mice. In particular, *MouseMove* resolved a ~3-fold reduction in mean speed (data not shown), a ~3-fold reduction in total distance travelled (Fig. 4c), and a ~70-fold increase in sedentary behaviour (stop time fraction; Fig. 4d) in MCAo-operated mice, relative to sham-operated mice.

### *MouseMove* vector analysis can be used to quantify laterality deficits in mice after experimental stroke

The unilateral brain damage characteristic of the MCAo model not only reduces general locomotor activity, but also produces laterality deficits such as circling^14^. Analysis of the OF behaviour using *MouseMove* clearly quantified this altered laterality, with MCAo-operated mice exhibiting a marked increase in turning bias (Fig. 5a,b) and tighter circling (Fig. 5c,d), relative to sham-operated mice. Thus, *MouseMove* provides a detailed quantification of altered OF behaviours following experimental stroke in mice.

### *MouseMove*’s region-of-interest (ROI) analysis can be used to quantify episodic recognition memory

The OF test can also be used to measure anxiety whereby increased fractional time spent close to the walls indicates increased anxiety^1^. Alternatively, apparatus (e.g. objects, cues, stimuli, mazes) can be placed into the OF to allow cognitive tests to be performed. In both these instances, movement needs to be analysed within defined sub-regions of the OF. Accordingly, we added a ROI function (see ROI tab in *MouseMove*.exe) to expand the utility of *MouseMove* and allow the quantification of certain cognitive assays.

To use the ROI function, videos are captured and handled in the same fashion as for the OF test. Once the video parameters and input files have been loaded into *MouseMove*’s GUI, one needs to click on the ‘ROI’ tab of the GUI and stipulate the centre and radius (in pixels) of each ROI. Up to 4 circular ROIs can be measured simultaneously. The fractional time spent within each ROI (i.e. ‘ROI fractions’) is then automatically measured and the results added to the saveable text file (Fig. 2i).

**Figure 6:**
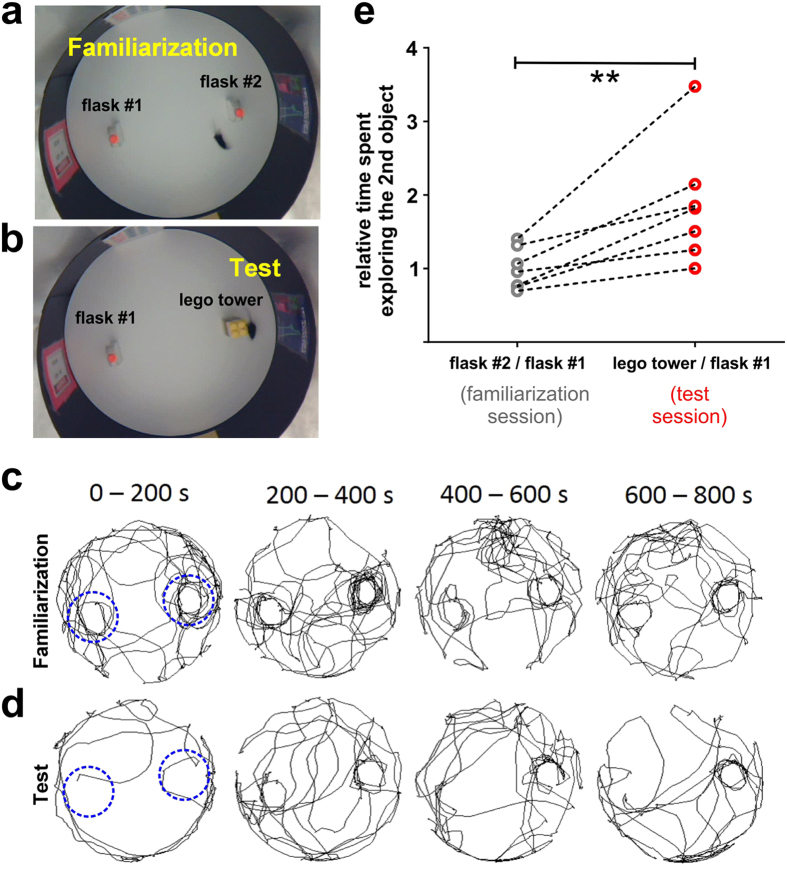
Analysis of novel object recognition using *MouseMove*’s region-of-interest function. (**a**,**b**) Video still of a representative mouse during familiarization (**a**) and test (**b**) sessions of the Novel Object Recognition (NOR) test. (**c**,**d**) Cumulative trajectories of the same mouse during familiarization (**c**) and test (**d**) sessions in 200 s intervals. The blue overlaid circles indicate that each ROI was centred on the stimuli (i.e. the lego tower and flask) and sized to be 30 pixel lengths in radius (i.e. a ~25 cm diameter circle). (**e**) Relative time spent exploring the 2^nd^ object (i.e. the lego tower in the test session or flask #2 in the familiarization session) versus the 1^st^ object (i.e. flask #1 in both the test and familiarization sessions). Dot points represent the mean value for individual mice (total of n = 7 mice). Lines connect the values for the same mouse across both NOR sessions. **p < 0.01 as determined by two-sided paired t-test.

To exemplify this ROI functionality, we modified the OF to perform the NOR test (see *Methods* for details). The NOR test is widely used to assess episodic memory^18^. It involves habituating a mouse in an OF containing two identical objects, then replacing one of the identical objects with a novel object (Fig. 6a,b). The time spent exploring the novel object, relative to the familiar object, is a gauge of episodic recognition memory^18^. As shown in Fig. 6c–e, the increased tendency of mice to explore the novel object was easily detectable using the ROI function of *MouseMove*. Based on these findings, *MouseMove* should not only enable semi-automated quantitative OF testing, but also allow digital analysis of cognitive tests such as the NOR.

## Discussion

The OF test was first described by Hall and Ballachey in 1932^19^. Today, the OF test is the canonical assay for comparative assessment of changes in locomotor activity. Here we demonstrate *MouseMove* to be an alternative, publicly available means of analysing the OF test in a semi-automated fashion. *MouseMove* has two downloadable components: an ImageJ macro and a separate program with a custom-built GUI. *MouseMove* utilises the pre-existing plugin, MTrack2^16^, to robustly track object movement. The *x,y* coordinates output file of the MTrack2 plugin is then fed into *MouseMove*’s GUI for quantitation of movement parameters including distance travelled, speed mean/S.E.M, fractional time spent stationary and indices of laterality (number of left turns, number of right turns, ratio of left:right turns [LRratio], turning offset and curvature). Lastly, *MouseMove* provides a visual overview of the cumulative tracking and graphical overview of distance travelled and speed distribution (Fig. 2h).

There are several important caveats with *MouseMove* when compared to the commercial OF analytical systems. For instance, whereas commercial systems offer an integrated package with an OF, movement recorder and analytic software, *MouseMove* users will be required to build their own OF, mount a video camera, install the ImageJ program and download our pre-processing macro and *MouseMove* .exe file. Building your own video-monitored OF, however, is straightforward and can be as simple as placing a ~0.4 m high circular wall on an appropriately coloured floor with an overhead webcam (Fig. 1). In addition, whilst *MouseMove* measures many of the same locomotion parameters as the ANY-MAZE and other commercial systems, it does not analyse higher-order events such as rearing or defecation and is incapable of distinguishing the rodent’s head from its tail.

In spite of these limitations, *MouseMove* offers numerous advantages to researchers who do not have access to a commercial OF analytical system. First, the script of *MouseMove* is publicly available and can therefore be customised. Once an OF is built, we show here that the *MouseMove*-based protocol can be used to provide accurate OF data in a semi-automated fashion, without the need for advanced equipment or specialist training. Second, the time taken for *MouseMove* to process and analyse video footage is relatively short compared to the time needed to perform these tasks manually (typically 10 min versus 3 h per OF test, respectively). *MouseMove* has undergone extensive in-house testing and (from March 2014-April 2015) has been used to quantify the locomotion of >150 mice (using high-throughput batch analysis; data not shown) across two different OFs in different research facilities. Third, we show that the ROI function expands the utility of *MouseMove* and allows analysis of OF-related cognitive tests such as the NOR test. Finally, while the studies described here have utilised *MouseMove* to analyse altered OF behaviours following experimental stroke in mice, this program could equally be suitable for the measurement of altered locomotion in other rodent models of brain injury/stimulation.

In conclusion, *MouseMove* is an open source, semi-automated and customisable means of performing the OF test. Accordingly, it should broaden usage of, not only the well characterised and popular OF test, but also other cognitive assays such as the NOR test.

## Methods

### Materials

Webcams used were the QuickCam E3560 or a HD Webcam C615 (Logitech; Lausanne, Switzerland). Videos were converted using Weeny Free Video Converter 2 version 2.1 (www.weenysoft.com). The video pre-processing utilised ImageJ 1.50a (National Institutes of Health, USA) and the MTrack2 plugin^16^ (designed by Nico Stuurman, Vale laboratory, University of California, CA, USA).

### Experimental animals

All animal procedures and methods were performed in accordance with the guidelines of the National Health and Medical Research Council Code of Practice for the Care and the Use of Animals for Experimental Purposes in Australia. All animal procedures and methods for this study were approved by the institutional Animal Ethics Committee (AEC), either AMREP AEC or Monash Animal Research Platform AEC. Experiments used adult male C57Bl/6 mice (aged 8–12 weeks). Mice were maintained under a 12 hour light/dark cycle with ad libitum access to food and water.

### Open Field (OF) Test

The OF test was performed according to the guidelines of Gould and colleagues^1^. In brief, OF testing was done in a quiet (~60 decibel) and dimly lit (~27 lux) room. Two OFs were designed to test *MouseMove.* The first OF (Fig. 1d) was used for the MCAo cohort (Figs 4 and 5) and featured a white melamine circular floor (0.79 m in diameter, Fig. 1a) and a black corrugated plastic wall (0.4 m in height, Fig. 1c). To minimize spatial cues, the OF was surrounded by a white opaque polyester curtain suspended from a bicycle wheel which, in turn, was suspended on top of semi-rigid fibreglass tent poles (Fig. 1b,c). A HD C615 webcam was fixed to the hub of the wheel ~1 m above the OF and connected via USB cable to a computer. The second OF (Fig. 1e) was used for mechanical calibration (Fig. 3) and for NOR testing (Fig. 6) and featured a white melamine circular floor (0.9 m in diameter, Fig. 1a), a black corrugated plastic wall (0.4 m in height, Fig. 1c) and was surrounded by a white opaque polyester curtain suspended from a rail affixed to the wall. A QuickCam E3560 webcam was fixed to the rail ~1.5 m above the centre of the OF and connected via USB cable to a computer (Fig. 1b,c). The OF was wiped with 70% (v/v) ethanol before each test to minimise olfactory cues. For testing, a mouse was placed in the centre of the OF and its movement recorded for 15 min. The mouse was then removed from the OF and a ~10 s video of the empty arena was captured. Note, we recommend that future users build an OF with similar dimensions and mount their video camera at a similar position as that shown in Fig. 1; this is because, whilst our software offers good flexibility in terms of arena size/camera placement, extreme departure (e.g. >2-fold) from our OF dimensions will require a compensatory change to be made to the *Preprocessing.*ijm and *MouseMove.*exe settings.

### Middle Cerebral Artery occlusion (MCAo) model of ischaemic stroke

The MCAo model was performed as described previously^20^. Mice underwent sham surgery or 1 h occlusion of the middle cerebral artery followed by 23 h of reperfusion and then OF testing.

### Novel Object Recognition (NOR) test

The NOR test was performed according to the guidelines of Leger *et al.*^18^ with minor modifications. In brief, four different laminated A4-sized pictures were equally spaced around the walls of the OF to facilitate spatial orientation (Fig. 6a). Two identical 225 ml tissue culture flasks (160 mm high, 90 mm wide, 38 mm deep) were filled with sucrose, parafilm-sealed then placed at opposite ends of the OF (~15 cm from the wall; Fig. 6a). A mouse was then allowed to explore the OF for 15 min whilst being video recorded (referred to as the ‘familiarisation’ session). 20 h later, one of the tissue culture flasks was replaced with a tower of lego pieces (tower was 170 mm high, 63 mm wide, 63 mm deep) and the same mouse was allowed to explore the OF for 15 min whilst being video recorded (referred to as the ‘test’ session). Note, after both familiarization and test sessions a ~10 s video of the empty arena was also captured.

### Video capturing, conversion and pre-processing

Videos are acquired at a spatial resolution of 640 × 480 pixels per frame (1~4 mm per pixel depending upon OF configuration) and a temporal resolution of 25 frames per second. Videos are then converted into MJPEG-compressed .avi files at full spatial and temporal resolution using Weeny Free Video Converter 2. The preprocessing macro (*Preprocessing*.ijm downloadable as Supplementary File 1) is then launched and the folder containing the converted .avi files is selected. Our pre-processing macro then spatially bins the videos into 320 × 240 before performing object segmentation/tracking whereby the ‘background video’ is subtracted from the ‘experiment video’. The subtracted video is then thresholded using the ImageJ’s ‘Minimum’ algorithm. To perform anti-aliasing, we applied ImageJ’s ‘erode’ and ‘dilate’ filters to the thresholded image. In our experience, anti-aliasing reduces artefacts (e.g. faeces) and is necessary for robust object segmentation/tracking. Object segmentation/tracking is then performed by feeding the anti-aliased video into the MTrack2 plugin. The output file generated by the MTrack2 plugin is then fed into *MouseMove*’s GUI for quantitation of movement parameters. Note, we recommend that future users capture/convert video footage with the same temporal and spatial resolution, because significant departure (e.g. >2-fold) from these video parameters will require a compensatory change to be made to the *Preprocessing.*ijm.

### *MouseMove* GUI design and algorithms

LabVIEW 12.0 Development System (National instruments, TX, USA) was used to build *MouseMove*.exe. *MouseMove* contains a ‘file import function’ which allows reading of the trajectory data file generated by our ImageJ macro and a ‘file export function’ for saving of the analysis results into a comma separated values text file. By default, *MouseMove* downsamples the tracking coordinates by a factor of 10 (i.e. effectively adjusting the tracking to 2.5 frames per second). The downsampled tracking coordinates are then used for analysis. To simplify analyses, it is assumed that mice only move in the forward direction (supported by our batch analysis of >150 mice where ~90% of movement occurs in the forward direction; data not shown).

For trajectory analyses: Let *n* be the total number of data points for the pre-processed trajectory. At *i*-th point (*t*_i_, *x*_i_, *y*_i_) from the pre-processed data, the distance increment is defined by Equation #1: 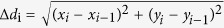. If Δ*d*_i_ = 0, ‘stop’ status was assigned to this time point. Stop time fraction was then determined by normalizing ‘stop’ time points over the *n*. The first 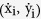 and second 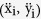 time derivatives of the displacement at each point 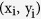 were calculated using the 2^nd^ order central discrete differentiation method with a window of three consecutive coordinates. For example, for i = 0, 1, 2, …, n – 1, 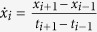. *x*_−1_ is the first element in initial condition, and *x*_n_ is the first element in final condition. The instantaneous speed is given by Equation #2: 
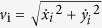
.

For region-of-interest analyses: The ‘ROI fraction’ calculated by *MouseMove* calculates the fractional time spent within each ROI. In other words, a ‘ROI1_fraction’ of 0.3 indicates that the tracked object spent 30% of its time within ROI 1. Note, each ROI is circular in shape and is defined by its centre and radius (coordinates which are manually entered into *MouseMove*’s ROI GUI).

For laterality analyses: A sliding window of 3 frames is used for all laterality measures. Within each sliding window, the vector across frames #2–3 is expressed relative to the vector across frames #1–2. The directionality of the mouse movement at *i*-th point is determined by the angle of the vector 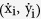 in Equation #3: 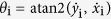. Thus, Equation #4: 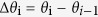 tells which direction the mouse will move in. Assuming a forward moving mouse cannot instantaneously turn over 90 degree, the decision rule of the directionality is: forward left (0 < Δ*θ*_i_ < π/2, Fig. 5a, *left*), forward right (−π/2 < Δ*θ*_i_ < 0, Fig. 5a, *right*), forward straight (Δ*θ*_i_ = 0) and backward (|Δ*θ*_i_|≥π/2). For each trajectory, the ratio of the left- against right-oriented time fractions (LRatio) was calculated (Fig. 5b, left y-axis) and |1- LRatio| is regarded as a further indication of movement laterality (Fig. 5b, right y-axis). To quantitate curvature radius at each point of the trajectory (Fig. 5c,d) we used Equation #5:


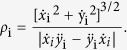


## Instruction for operating ***MouseMove***

**Prerequisites:**

1. A computer with a Microsoft Windows operating system.
2. ImageJ 1.50a (Fiji) ; can be downloaded from http://fiji.sc/Downloads
3. *Preprocessing.*ijm can be downloaded via the link for Supplementary file 1.
4. *MouseMove*.exe can be installed after downloading via the link for Supplementary file 2. Users may be prompted to install LabVIEW if not already installed on their computer.

**Step-wise instructions:**

1. Create a new folder which contains at least one experiment video file and one background video file (.avi file format with a spatial resolution of 640 × 480 and a temporal resolution of 25 frames per second). The same name for corresponding experiment and background videos should be used, but with the suffix ‘empty’ added to the background video file name. Note, multiple videos can also be placed in the same folder for batch analysis, however, the names of the corresponding experiment/background videos must be paired (e.g. experiment #1 filename = ‘MCAo mouse a.avi’, background #1 filename = ‘MCAo mouse a empty.avi’, experiment #2 filename = ‘MCAo mouse b.avi’ and background #1 filename = ‘MCAo mouse b empty.avi’).
2. Open the macro *Preprocessing.*ijm in ImageJ.
3. Click ‘Run’ and the interface will prompt you to identify where the video-containing folder is located. The macro will preprocess all videos in the folder and save the output trajectory data as ‘TkResults …txt’ files. Note: because of ImageJ’s memory restrictions, videos are segmented into 5000 frame lengths, and therefore the output files are named by combining the original video name with the frame range (e.g. ‘TkResults_MCAo mouse a_1-5000.txt’).
4. Open the *MouseMove*.exe and click the yellow ‘folder open’ button to import one TkResults file. *MouseMove* will then automatically recognise and stitch together the other segments from the same experiment. After this, *MouseMove* automatically displays its analysis results.
5. To analyse different frame range, recalibrate OF spatial and temporal parameters, downsample the video and stipulate the centre and radius of an ROI (in the ROI tab) simply change these parameters in *MouseMove*’s GUI and click ‘Update’.
6. To start a new analysis of other preprocessed data files, simply click the yellow ‘open folder’ icon and import a new file-of-interest. The software will automatically update the results.
7. Click the ‘Save’ button to save the analysis result as a comma separated values text file (.csv) within the original video-containing folder.

Note, example ‘background’ and ‘experiment’ video files can be downloaded as Supplementary files 3 and 4, respectively, and used to test the proper functioning of *Preprocessing*.ijm macro and *MouseMove*.exe.

### Statistical Analyses

Statistical analyses were performed using GraphPad Prism® 6.04 (GraphPad Software, Inc.). Results are expressed as mean ± s.e.m. For each cohort, the number of independent experiments and the statistical test employed is indicated in the respective legend. p < 0.05 was considered to be statistically significant.

## Additional Information

**How to cite this article**: Samson, A. L. *et al.* *MouseMove*: an open source program for semi-automated analysis of movement and cognitive testing in rodents. *Sci. Rep.* **5**, 16171; doi: 10.1038/srep16171 (2015).

## Supplementary Material

Supplementary Information

Supplementary File 1

Supplementary File 2

Supplementary File 3

Supplementary File 4

Supplementary Video 1
