# Supplementary material for: MouseMove: an open source program for semi-automated analysis of movement and cognitive testing in rodents
Source: Sci Rep. 2015 Nov 4;5:16171. doi: 10.1038/srep16171 (PMC4632026; doi:10.1038/srep16171)
Supplement: Supplementary Information [file srep16171-s1.pdf]

Supplementary Material

For manuscript entitled: **"*MouseMove*: an open source program for semi-automated analysis of movement and cognitive testing in rodents."** by Samson AL†, Ju L†, Kim HA, Zhang SR, Lee JAA, Sturgeon SA, Sobey CG, Jackson SP and Schoenwaelder SM (2015).

List of additional supplementary files

Supplementary Video 1 | OF testing of sham-operated and MCAo-operated mice.

Video compares footage of a sham-operated and a MCAo-operated mouse before and after processing with *Preprocessing*.ijm.

Supplementary File 1 | *Preprocessing*.ijm macro (requires unzipping).

Supplementary File 2 | *MouseMove*.exe installer (requires unzipping).

Supplementary File 3 | Example .avi 'background' video for testing *MouseMove*.

Supplementary File 4 | Example .mp4 'experiment' video for testing *MouseMove*. Note, one needs to convert this file into .avi format before using it to test *MouseMove*.
